# Antegrade Aorto-Mesenteric Bypass Using a Prefabricated Bovine Pericardium Tube Graft for the Treatment of Chronic Mesenteric Ischemia Complicated With Intestinal Necrosis and Biliary Peritonitis

**DOI:** 10.7759/cureus.57530

**Published:** 2024-04-03

**Authors:** Alexander T Daskalov

**Affiliations:** 1 Vascular Surgery, Acibadem City Clinic Tokuda Hospital, Sofia, BGR

**Keywords:** compromised operative field, tube graft, chronic mesenteric ischemia, bovine pericardium, aorto-mesenteric bypass

## Abstract

Chronic mesenteric ischemia (CMI) is a vascular disorder primarily caused by atherosclerosis, resulting in intestinal ischemia. While endovascular treatment has become the primary modality for most patients, open mesenteric revascularization remains crucial for complex cases. We present a case of CMI in a patient with critical ischemia, leading to small bowel necrosis, where the endovascular recanalization failed and a surgical approach was needed. A supraceliac antegrade aortomesenteric bypass was performed, and successful revascularization of intestinal circulation was achieved. A novel prefabricated bovine pericardium tube was used as a graft, and the bypass was placed behind the pancreas to ensure maximal isolation from the contaminated abdominal cavity. Despite the intestinal revascularization, in the early postoperative period, the overall condition of the patient worsened with obvious signs of peritonitis. The second look operation revealed a ruptured gallbladder with severe biliary peritonitis, likely caused by the preceding splanchnic ischemia. A cholecystectomy, lavage, and drainage were performed. No further intestinal necrosis was observed, and the bowel passage was restored with latero-lateral jejuno-lejunostomy. The follow-up of the patient showed no signs of graft infection. Despite the complications, the patient's postoperative period was stable, and he was discharged on day sixteen. Regular follow-ups confirmed an excellent patency of the bypass.

## Introduction

Chronic mesenteric ischemia typically arises from occlusive atherosclerosis impacting at least two of the three mesenteric arteries. Additional contributing factors may include vasculitis, fibromuscular dysplasia, neurofibromatosis, dissection, trauma, embolization, and distal thoracic or abdominal aortic coarctation. The main goals of treatment are to prevent bowel infarction, relieve symptoms, and restore normal weight [1].

The management of chronic mesenteric ischemia has developed significantly over the past twenty years. Initially, endovascular treatment was introduced as a substitute for bypass surgery, particularly in older individuals or those at high risk. However, it has become the main approach for most patients, regardless of their surgical risk level. Nonetheless, open mesenteric revascularization through bypass surgery or, in rare cases, endarterectomy remains crucial for treating patients with more extensive disease presentations, such as long-segment or complete blockages, multiple sequential lesions, and significant calcification [1,2].

We present a case of severe chronic mesenteric ischemia, the decision-making process involved, and preliminary results obtained through the use of a bovine pericardial graft as a bypass conduit in a compromised operative field with a high probability of graft infection.

## Case presentation

**Figure 1:**
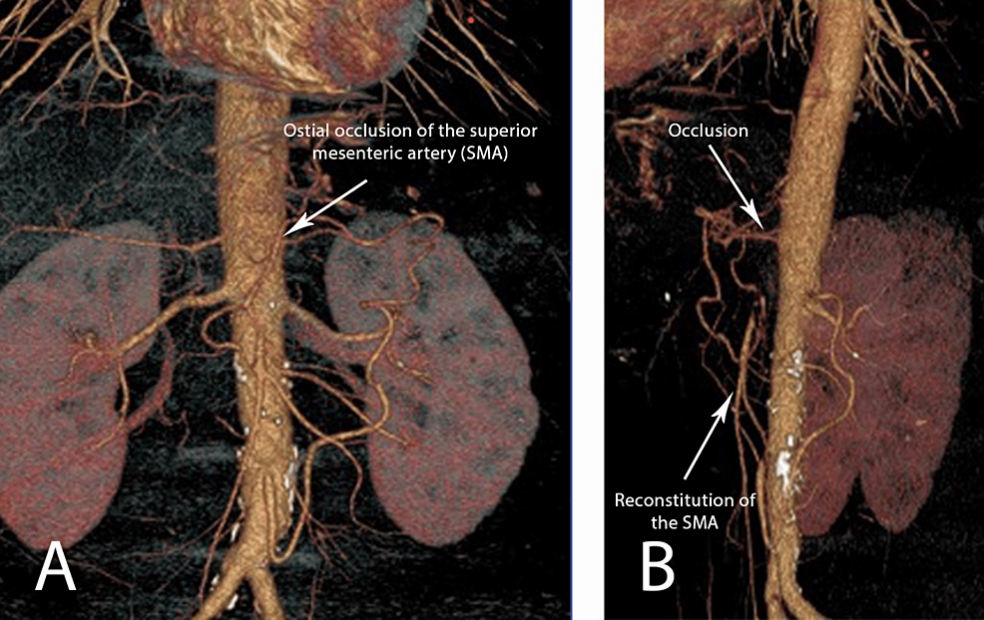
Preoperative CT-Angiogram showing total ostial occlusion of the superior mesenteric artery А-Frontal view; B-Side view; SMA: superior mesenteric artery

We report a case of a 54-year-old male who presented at the emergency department with a four-month history of worsening abdominal pain, which intensified during the last five days before admission. The patient lost 10 kilograms over the four months. He had the following comorbidities: hiatal hernia, pangastritis, and peptic ulcer. There were no signs of systemic atherosclerosis or markers indicating systemic vasculitis upon arrival at the hospital. A computer tomography angiogram (CTA) scan showed a long flush occlusion of the superior mesenteric artery (SMA). The celiac trunk was completely occluded from the ostium with no reconstructable target vessel distal to the thrombosis, but a patent inferior mesenteric artery (IMA) was present (Figure 1).

The CTA showed no signs of bowel ischemia or pathological findings in the gallbladder. Because of the continuous abdominal pain, an attempt for immediate transbrachial endovascular recanalization of the SMA was made. Despite our efforts, we were unable to perform endovascular treatment-no stumps of SMA or the celiac trunk were visible, and the intraoperative angiogram showed no run-off vessels.

The patient's symptoms didn't improve in the next few hours despite a proactive implementation of the treatment dose of heparin and vasoactive agents. Clinical signs of peritonitis were observed - diffuse abdominal tenderness, vomiting, and abdominal musculature rigidity. In this rapidly worsening situation, an urgent surgical revascularization was necessary.

A midline laparotomy incision was made, and the thorough exploration of the abdomen revealed pathologic signs of peritonitis and a heavily ischemic part of the jejunum with a total length of around 25 cm. Multiple smaller ischemic regions throughout the small intestine were present as well. No perforation was found at this stage. Taking into account the hostile terrain and the need for intestinal resection, the use of synthetic grafts was not desirable. Therefore, we performed an antegrade aortomesenteric bypass using a prefabricated bovine pericardial tube graft (Biointegral Surgical No-React® (Biointegral Surgical Inc), Mississauga, ON, Canada), which was available off-the-shelf.

The attention was directed towards the exposure of the distal thoracic aortic inflow source. The triangular ligament of the left lobe of the liver was cut, and moist laparotomy packs were placed to safeguard the liver tissue. Access to the lesser sac was gained by cutting the gastrophrenic ligament. The esophagus was moved to the left, and further exposure of the aorta was accomplished by dividing the diaphragmatic crura and the median arcuate ligament. Once the supraceliac aorta was meticulously dissected and prepared for the proximal anastomosis, the surgical focus temporarily shifted to the mid-abdomen by raising and moving the transverse colon upward. The duodenum's small intestine and the fourth segment were pulled to the right. The peritoneal membrane was incised, and the patent distal segment of the SMA was isolated for distal anastomosis. Proximal and distal anastomoses were sutured end-to-side, and the graft was tunneled behind the pancreas. At the end of the procedure, there was no visible part of the graft in the abdominal cavity.

After restoring blood flow, an immediate change in intestinal circulation was observed, marking the extent of intestinal necrosis. A general surgeon carried out resection of the ischemic intestine following, without restoring the bowel passage and intending to perform a second look after 48h. The patient was stable throughout and after the procedure. He was extubated immediately and transferred to the ICU for monitoring.

In the following 24 hours, the patient's overall status worsened despite the revascularization and the resection of the ischemic bowel. His abdominal pain reappeared, and he quickly developed the clinical presentation of an acute abdomen. Abdominal guarding in the right flank was present.

An emergency second-look operation was done. We stumbled upon a ruptured gallbladder and the presence of biliary peritonitis, likely due to the splanchnic ischemia preceding the revascularization. Cholecystectomy, ligature of cystic duct, lavage, and drainage were performed. There were no additional necrotic areas in the intestine, and the bowel passage was restored with latero-lateral jejuno-jejunostomy. Abdominal passive drainage was held for ten days thereafter. The patient had a short polyuria episode followed by normuria. Overall, the postoperative period was stable, and the patient was discharged on day sixteen after the last surgery.

**Figure 2:**
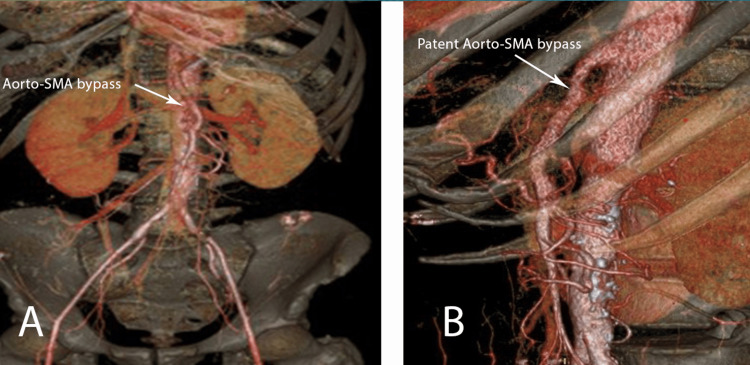
Post-operative CT-Angiogram showing a patent reconstruction using a bovine pericardium prosthesis A-Frontal view; B-Side view; SMA: superior mesenteric artery

A follow-up CT scan, performed one month after the patient was discharged, showed an excellent patency of the bypass (Figure 2). The patient attended regular follow-up checks for one year without further complications or symptoms. The patency was monitored with a duplex scan.

## Discussion

Mesenteric arterial insufficiency, although rare, can lead to persistent abdominal pain. Patients typically experience prolonged symptoms before receiving a definitive diagnosis and treatment. Due to this delay, patients may suffer from poor nutrition and be deemed at increased risk for surgery, particularly when facing an acute-on-chronic occlusion. Nowadays, the vast majority of patients suffering from chronic mesenteric ischemia are treated by endovascular means [1,2]. Open revascularization remains crucial for complex cases of CMI when an endovascular attempt fails. The surgical bypass offers a low mortality rate and excellent long-term primary patency rates. Open surgery might be the preferred approach when faced with challenging anatomical conditions such as flush or extensive blockages, severe calcification, narrow vessels, tandem lesions, or occluded stents, and in younger patients with nonatherosclerotic lesions [3,4].

Retrograde percutaneous recanalization of SMA, either via IMA or celiac trunk, has also been reported lately with promising results [5]. This technique requires special equipment and skills, is time-consuming, and thus may not be feasible in an emergency. Larger studies are needed for further conclusions.

Another viable option is hybrid retrograde recanalization of SMA using intraabdominal exposure of SMA distal to the occluded portion (ROMS-retrograde open mesenteric stenting). There are several publications regarding this technique. Jean N. Sénémaud et al. presented a cohort of 37 patients treated by ROMS. The technical success rate was 89%, and retrograde recanalization was impossible in four cases. ROMS failures were more frequently observed in patients presenting with prolonged occlusion of the superior mesenteric artery. Out of the 33 patients who underwent ROMS, the one-year primary patency and one-year assisted primary patency rates were 84.54% and 92.4%, respectively. At one year, the rate of freedom from re-intervention was 61.14%. One of the most commonly reported intra-operative complications during the ROMS procedure was the inability to re-enter the true aortic lumen, as noted in other studies [6]. Such occurrences necessitate bailout bypass surgery, potentially leading to extended operative time and increased duration of bowel ischemia on already compromised bowel, which may result in higher mortality rates [7].

Regarding the endovascular technique in particular, according to the clinical practice guidelines from the Society for Vascular Surgery published in 2020, balloon-expandable covered intraluminal stents seem to be the optimal stent choice and likely afford the same advantages as reported in other anatomic locations [8]. Covered stents were found to have fewer restenosis and re-interventions than bare metal stents, especially in acute-on-chronic thrombosis with a probability of a fresh thrombus. Although extremely rare, stent-graft infections could also occur due to intestinal necrosis and septic conditions [9].

In the case reported here, the initial endovascular approach failed to bring any results, and the sheer amount of ischemia present in the small bowel solidified the choice of open surgery as the most appropriate revascularization method for this patient.

In recent years, there has been significant advancement in open mesenteric revascularization, making it the preferred standard approach [2]. Recent studies have shown that mesenteric bypass can be safely carried out by experienced surgeons with mortality rates comparable to endovascular treatment [1]. Kruger et al. showed a primary graft patency of 92.4% at 60 months. No notable discrepancies were found in graft patency rates across various configurations or conduits for bypass grafts. Even though patients with chronic mesenteric ischemia (CMI) requiring mesenteric revascularization are usually seen as having a high risk for surgery, their reported perioperative mortality stands at only 2.6% [3]. In cases of compromised operative, field reports have shown excellent results of patency and survival when using auto venous reconstruction - with some advantages when using superficial femoral versus saphenous veins because of the larger diameter and thicker wall [10]. Vein harvesting is time-consuming and adds additional surgical load for the septic patient.

Another option is using bovine pericardium, mostly available as a patch, which can be sutured to form a tubular graft. We have used this technique to treat patients with infected prosthetic grafts with excellent results. Modifying the tube from the patch is also a time-consuming procedure and is thus not appropriate in urgent conditions.

In the presented case, a prefabricated Biointegral vascular graft (7 mm, 40 cm) was available off-the-shelf (BioIntegral Surgical Inc, Mississauga, Ontario, Canada). This graft is created from bovine pericardium, which undergoes multiple cleaning and rinsing cycles before being cross-linked with low-pressure and low-concentration glutaraldehyde solutions. Once cross-linking is confirmed, the tissue is rolled and sealed into a tube or Y-prosthesis using three layers of manual suturing. Two continuous layers secure the graft to prevent leakage, while the third layer is intermittently interrupted every 2 mm to allow for adjustment as needed. Following this, the graft is sterilized using a high aldehyde concentration and then thoroughly rinsed with saline and heparin to remove any remaining aldehyde residues through the 'No-React' procedure. Before application, the graft is only moistened with saline and is then ready for use.

In our case, the main reasons to favor this graft type were its immediate availability and proclaimed infection resistance even for use in infected areas. There are some studies regarding the usage of this prefabricated graft. Rojas M et al. analyzed clinical data of patients surgically treated for vascular graft infections in aortoiliac position for two years. This study included 9 cases [11] and showed an acceptable patency and free of reinfection rates. Burghuber et al. have presented the largest series to date from two vascular centers (21 patients) using the prefabricated biological graft to replace the infrarenal aorta. They found that primary patency rates at year 1 and year 2 were 94% and 86%, and the assisted primary patency rate was 94% and 94%, respectively [12]. Reinfection was found in two patients. A pseudoaneurism formation was found in one patient four years after implantation. Pseudoaneurysms were rarely observed in the previous series using physician-sutured bovine grafts [13]. This data supports the rationale for using this type of graft, even in younger patients, as in the present case report.

There has been significant discussion previously regarding the method of open mesenteric revascularization (such as endarterectomy versus bypass), the design of the bypass graft (either antegrade or retrograde), and the optimal number of vessels to be revascularized (single versus multiple). Selecting the method is up to the surgeon, depending on the lesions. Antegrade bypass is commonly chosen because the supraceliac aorta is usually unaffected by atherosclerosis. Conversely, the retrograde technique provides easier access to the in-flow vessel [4]. We opted for the anterograde bypass technique, taking as few risks as possible in the already compromised operative field, tunneling the bypass behind the pancreas, and ensuring all of the graft material was covered by healthy tissue and isolated from potentially infected areas. Another reason for choosing the antegrade technique was the straight layout of the bypass in this position. This allowed us to avoid possible kinking, which the used Biointegral graft is prone to, mainly because of the 3-layer suture lines, making the seam relatively rigid [12]. While most surgeons concur that revascularization should primarily target the SMA, there remains significant debate regarding the preference between single- and multiple-vessel revascularization. In the presented case, the celiac trunk was completely occluded with no distal target vessels, and there was no option for meaningful revascularization. Therefore, a single vessel revascularization was applied.

The good collaboration with general surgeons to perform an adequate bowel resection was of great importance. In this clinical case, the resection was made after the restoration of blood flow, when the bypass was already completely covered by healthy tissue and the boundary between bowel necrosis and the viable intestine was better detectable. We performed blind sutures of the two ends of the intestine, awaiting the second-look operation after 48 hours. As already described, in the next 24 hours, the condition of the patient worsened, presenting obvious signs of peritonitis. The intestine was found viable without further necrosis during the second look. A ruptured gallbladder was observed, causing severe biliary peritonitis. In the literature, this condition is called acute acalculous cholecystitis (AAC). The reason for this complication was perhaps ischemic necrosis of the gallbladder before blood flow restoration. In the context of chronic mesenteric ischemia (CMI), acute acalculous cholecystitis (AAC) has been infrequently documented historically, as the primary concern with CMI has typically been acute mesenteric ischemia and subsequent bowel infarction. However, Savoca et al. presented two cases where AAC arose as a secondary consequence of mesenteric graft occlusion in CMI-treated patients [14], while Koea et al. reported a case of AAC in a patient later diagnosed with CMI [15]. Presently, AAC is recognized as a multifaceted process, primarily involving bile stasis and ischemia.

Ischemic damage is pivotal in AAC's development due to the terminal nature of the cystic artery, rendering the gallbladder susceptible to low-flow conditions [16]. Several case reports have also highlighted AAC occurrences preceding mesenteric infarction in acute mesenteric ischemia cases, suggesting AAC as an indicator of critical ischemia and impending mesenteric infarction [16]. Although AAC complications have not been encountered in our practice with CMI patients, this case underscores the significance of visceral ischemia as a predisposing factor for AAC, which could potentially complicate CMI cases. This also underscores the appropriate position of the bypass (outside the abdominal cavity) and the right choice of infection-resistant graft material. Despite the few publications available regarding using prefabricated bovine pericardium as a bypass conduit, we believe it served a great benefit in the current case. Self-made animal pericardial tubes have been used for many years in infected aortic graft surgery, showing superior biocompatibility and reduced infection rates to prosthetic material [17,18]. We believe this is the first ever documented case of this type of conduit being used for an aorto-mesenteric bypass reconstruction.

Given the already compromised and potentially infected operative field, our reconstruction was not affected by the infection and was deemed a reliable alternative to synthetic grafts such as Dacron and PTFE. In our clinical case, the use of bovine pericardium proved to be effective in this hostile abdomen situation. The bovine pericardial tube graft reconstruction showed excellent patency in the first year.

## Conclusions

In summary, open mesenteric revascularization remains crucial for addressing complex cases of CMI. Using bovine pericardial graft as a bypass conduit in challenging surgical scenarios has demonstrated promising initial outcomes, suggesting it is a viable substitute for synthetic materials. Additional studies involving larger cohorts and longer observation periods are required to confirm these findings and establish the effectiveness of bovine pericardium grafts in mesenteric revascularization.
